# Zinc-lysine Supplementation Mitigates Oxidative Stress in Rapeseed (*Brassica napus* L.) by Preventing Phytotoxicity of Chromium, When Irrigated with Tannery Wastewater

**DOI:** 10.3390/plants9091145

**Published:** 2020-09-04

**Authors:** Ihsan Elahi Zaheer, Shafaqat Ali, Muhammad Hamzah Saleem, Muhammad Arslan Ashraf, Qurban Ali, Zohaib Abbas, Muhammad Rizwan, Mohamed A. El-Sheikh, Mohammed Nasser Alyemeni, Leonard Wijaya

**Affiliations:** 1 Department of Environmental Sciences and Engineering, Government College University Allama Iqbal Road, Faisalabad 38000, Pakistan; ihsankhanlashari@gmail.com (I.E.Z.); zohaib.abbas83@gmail.com (Z.A.); mrizwan@gcuf.edu.pk (M.R.); 2 Department of Biological Sciences and Technology, China Medical University, Taichung 40402, Taiwan; 3 MOA Key Laboratory of Crop Ecophysiology and Farming System Core in the Middle Reaches of the Yangtze River, College of Plant Science and Technology, Huazhong Agricultural University, Wuhan 430070, China; 4 Department of Botany, Government College University Faisalabad, Faisalabad 38000, Pakistan; marslanashraf@gcuf.edu.pk; 5 Key laboratory of Plant Pathology, College of Plant Science & Technology, Huazhong Agricultural University, Wuhan 430070, China; rattarqurban@hotmail.com; 6 Department of Botany and Microbiology, College of Science, King Saud University, 11451 Riyadh, Saudi Arabia; melsheikh@ksu.edu.sa (M.A.E.-S.); mnyemeni@ksu.edu.sa (M.N.A.); leon077@gmail.com (L.W.)

**Keywords:** antioxidant enzymes, growth, heavy metals, oil seed crop, micronutrient-amino chelates

## Abstract

Contamination of soil and water with metals and metalloids is one of the most serious problems worldwide due to a lack of a healthy diet and food scarcity. Moreover, the cultivation of oilseed crops such as rapeseed (*Brassica napus* L.) with tannery wastewater could contain a large amount of toxic heavy metals [e.g., chromium (Cr)], which ultimately reduce its yield and directly influence oilseed quality. To overcome Cr toxicity in *B. napus*, a pot experiment was conducted to enhance plant growth and biomass by using newly introduced role of micronutrient-amino chelates [Zinc-lysine (Zn-lys)], which was irrigated with different levels [0% (control), 33%, 66%, and 100%] of tannery wastewater. According to the results of present findings, very high content of Cr in the wastewater directly affected plant growth and composition as well as gas exchange parameters, while boosting up the production of reactive oxygen species (ROS) and induced oxidative damage in the roots and leaves of *B. napus*. However, activities of antioxidants initially increased (33% of wastewater), but further addition of tannery wastewater in the soil caused a decrease in antioxidant enzymes, which also manifested by Zn content, while the conscious addition of wastewater significantly increased Cr content in the roots and shoots of *B. napus*. To reduce Cr toxicity in *B. napus* plants, exogenous supplementation of Zn-lys (10 mg/L) plays an effective role in increasing morpho-physiological attributes of *B. napus* and also reduces the oxidative stress in the roots and leaves of the oilseed crop (*B. napus*). Enhancement in different growth attributes was directly linked with increased in antioxidative enzymes while decreased uptake and accumulation of Cr content in *B. napus* when cultivated in wastewater with the application of Zn-lys. Zn-lys, therefore, plays a protective role in reducing the Cr toxicity of *B. napus* through an increase in plant growth and lowering of Cr uptake in various plant organs. However, further studies at field levels are required to explore the mechanisms of Zn–lys mediated reduction of Cr and possibly other heavy metal toxicity in plants.

## 1. Introduction

The rapid industrial and urbanization caused by the population’s overly growing threat posed a threat to humanity by air, water, and land contamination with different heavy metals [1]. Tanneries have played a significant role in soil and water contamination among industries. Farming practices utilize approximately 70% of the water drains, and farmers should use domestic or urban wastewater as a source of water in times and regions of water scarcity [2]. Due to the scarcity of fresh water, wastewater cultivation became a common practice in arid and semi-arid areas, which has serious impacts on plants and human health [3]. The two major cities with a large number of tanneries in Pakistan are Sialkot and Kasur. In these areas, different factories emit large amounts of untreated wastewater and contaminate the region’s freshwater resources [4]. Pakistan is also now facing a severe shortage of fresh water due to a large number of factories and manufacturers, and thus farmers often use industrial wastewater to cultivate fields [2]. Agricultural soils mainly contain low heavy metals and soil contamination with these toxic pollutants through fertilization or tannery wastewater irrigation [5]. The wastewater does, however, contain a low content of heavy metals; due to metal uptake and accumulation, the plant had higher metal contents in their body organs [6]. Chromium (Cr) is the most significant among different heavy metals found in tannery wastewater due to its large concentration [7]. Cr in abundance is harmful to plants; plant growth and composition are directly affected, with significant impacts on crop quality and decreased soil mineral uptake [8]. In addition, excess Cr has also been caused by the generation of extreme amounts of reactive oxygen species (ROS) and also the alteration of the many antioxidative enzymes in plants [3,9]. Therefore, the exploration of various technologies and different management methods is extremely important to restrict the accumulation of Cr by several plants in soil polluted by Cr and consequently to reduce the human health risks associated with Cr toxicity.

Recently, various studies have indicated the imperious role of different amino acids in alleviating heavy metal stress in the various plants [10]. Zinc (Zn)-chelated fertilizer with amino acid, like lysine (lys), is documented to boost crop yield and production [3,11]. Combining Zn with lysine significantly improves morpho-physiological attributes of the plants and nutrient uptake [9]. In addition, Zn-lys has also documented increased use of essential nutrients to increase the gas exchange attributes and improve the photosynthetic apparatus of the plants [11]. It has also been noted previously that the combination of essential micronutrients with lysine decreased the salinity effect in tomato plants and increased plant growth and crop yield [12]. Earlier research also indicated that exogenous Zn supplementation with lysine decreased Cr toxicity in many important agronomic plants, including wheat [9] and Cd toxicity in rice [11].

Oil rape (*Brassica napus* L.) is the world’s second most important source of vegetable oil and has the capacity to accumulate in the edible portion of toxic heavy metals from heavy-metals soil polluted by irrigation of wastewater [2,13]. *B. napus* is cultivated extensively around the worldwide and the stress relief of heavy metals has also been widely investigated in different environmental conditions [2,14]. However, it showed severe damage in different organs when cultivated in the soil, which was polluted with various heavy metals in terms of grain quality and widespread productivity [14]. The irrigation of *B. napus* with wastewater is a very common practice in the world due to the availability of fresh water [2,14]. The threat of public exposure to these toxic pollutants occurs rapidly when contaminated food crops are being consumed in agricultural fields irrigated by wastewater. Furthermore, *B. napus* is also used to cultivate in metal-polluted soil and showed resistance against heavy metals stress conditions [15,16]. In addition, various chelators or phytohormones have been used exogenously to increase the phytoextraction of heavy metals with *B. napus* to increase the metal uptake/accumulation [14,15,17,18]. Although very large amounts of heavy metals in plant organs seriously affected the quality of their oilseeds and could not be used for oilseed rape. The uniqueness of *B. napus* due to its high biomass production and tolerance towards Cr can be valuable traits; however, sufficient information is not available regarding Cr tolerance, gas exchange traits, oxidative stress and antioxidative defense system, and Cr accumulation, when grown as oilseed rape. In order to improve plant growth and composition by the foliar application of Zn-lys, when cultivated on different levels of tannery wastewater (Cr-polluted soil), the current experiment was designed. The results from this study would add to our knowledge about (i) the effect of different levels of tannery wastewater in the soil on plant growth and biomass, (ii) photosynthetic pigments and gas exchange attributes, (iii) oxidative stress and antioxidative defense system, and (iv) Zn and Cr uptake and accumulation in various parts of the plants, when supplied externally with the foliar application of Zn-lys. To the best of our knowledge, this study is among the few studies that focus on the metal tolerance among oilseed crops in order to investigate their suitability for metal-contaminated sites with the exogenous application of micronutrient chelation with amino acid. Findings from the present study will add to our understanding of the mechanism of Cr tolerance in *B. napus*, when externally supplied with the foliar application of Zn-lys.

## 2. Materials and Methods

### 2.1. Collection and Analysis of Wastewater and Soil

Clay loam sand used in this analysis was collected from the botanical garden of the University of Punjab Lahore Pakistan (31.4015° N, 74.3070° E) from an average depth of 0–20 cm. The 2 mm sieve was used to separate mud and debris from the dried soil intentionally. A comprehensive analysis of organic soil content was performed by the process Walkley and Black [19] described. For the precise size of the soil elements, the hydrometer was used [20]. The physiochemical characteristics of the sand under study are given in Table 1. Tannery wastewater used for the pot experiment was collected from the tannery industries based in Kasur, Punjab, Pakistan. The physicochemical properties of tannery wastewater used in the presented study were estimated according to the set methods of Apha [21]. Comprehensive details of the major characteristics of tannery wastewater used in this experiment are presented in Table 2. The same soil with the tannery wastewater we already used in our previous study by Zaheer et al. [3].

### 2.2. Pot Experiment

Healthy and mature seeds of rapeseed (*Brassica napus* L.) were collected from Ayub Agriculture Research Institute Faisalabad, Pakistan. Ten seeds of *B. napus* were carefully sowed in experimental pots filled with approximately 5 kg of soil. Seeds were surface-sterilized with 10% (*v/v*) commercial bleach for 15 min, followed by thorough washing in distilled water. Thirty days old baby plants were applied with foliar application of Zn-lys (10 mg L^−1^) alongside with various levels of tannery wastewater (0%, 33%, 66%, and 100%) applied gently. Tannery wastewater was added in the pots, and all pots were given 0%, 33%, 66%, and 100% wastewater on the basis of the treatments when needed with other intercultural operations such as weeding. Moreover, with the addition of tannery wastewater (at least) once a week, we also sprayed Zn-lys regularly, on the basis of demand, on whole plant parts. The experiment was arranged in a way that 8 treatments were as follows: (Ck) Cr 0%, Zn-lys 0 mg L^−1^ (T1) Cr 0%, Zn-lys 10 mg L^−1^ (T2) Cr 33%, Zn-lys 0 mg L^−1^ (T3) Cr 33%, Zn-lys 10 mg L^−1^ (T4) Cr 66%, Zn-lys 0 mg L^−1^ (T5) Cr 66%, Zn-lys 10 mg L^−1^ (T6) Cr 100%, Zn-lys 0 mg L^−1^ (T7) Cr 100%, Zn-lys 10 mg L^−1^. The total time duration of the treatments was 60 days, and we used all the samples of the plants from each treatment for the analysis of morpho-physiological traits of the plants.

A hand sprayer was used for the exogenous supplementation of Zn-lys to the *B. napus*. In the whole experiment, we used a 4 L volume of Zn-lys for all the pots/plants, which were applied at different times of the experiment. Every time when we applied tannery wastewater, the plant was also sprayed exogenously with the application of Zn-lys. The experiment was executed in 3 replications of each treatment, and 5 plants were grown in a single pot. The present experimental work was conducted in the wire house Department of Botany, the University of the Punjab Lahore, Pakistan, in an open environment, protected from human and animal interactions. The rainfall was controlled, or plants were protected from rainfall by covering the whole wire house with a plastic sheet. Fertilizers such as urea (250 mg/pot), diammonium phosphate (100 mg/pot), and potassium sulfate (50 mg/pot) were applied to each pot to avoid macronutrient deficiency in the plants. The pots used in this study were rotated regularly in order to avoid environmental effects on the plants.

### 2.3. Plant Harvesting

Plants of *B. napus* were carefully rooted-up after 30 days of experimental treatment (precisely 60 days of the whole experiment) and washed gently with the help of distilled water to eliminate the aerial dust and deposition of soil and silt particles. All the harvested plants were divided into 2 parts, i.e., roots and shoots, to study different biological traits. Plant height (whole plant length) was measured straightway after the harvesting using a measuring scale, and the number of leaves per plant was also counted after it. The number of leaves and leaf area was measured, and the fresh and dry biomass of the leaves were measured, and then the remaining were analyzed for Cr and Zn content from the shoots, after mixing with stems and other shoots parts. All the plant samples (after harvest) were attentively washed with the help of de-ionized water, and after that, oven-dried at 70 °C (for 3 days) and then grounded to a very fine powder to complete further investigation.

### 2.4. Analysis Items and Methods

For all physiological and Cr and Zn determination from different parts of the plants, we used 3 replications for each sample. The mass of the leaf or root samples was collected/taken as recommended in different protocols, which are mentioned below. All the physiological traits were performed using fresh biomass of the plants, which was stored in the refrigerator (−80 °C). Total chlorophyll content (Chl *a* + Chl *b*) and carotenoid content were measured by following Nagata and Yamashita [23]. In 80% acetone, 1 g fresh plant of leaves was pulverized using a pestle and mortar, and the resolution was centrifuged at 3000 rpm for 10 min. Calculations were made using the following formulas:

Chlorophyll *a* (mg/g FW) = 0.999 A_663_ − 0.0989 A_645_

Chlorophyll *b* (mg/g FW) = 0.328 A_663_ + 1.77 A_645_

Carotenoid (mg/g FW) = 1000 A_470_ − 2.270 C_a_ − 81.4 C_b_/227

where C_a_ is chlorophyll *a* content and C_b_ is chlorophyll *b* content

While at a bright sunny day (9:00 a.m.–11:00 a.m.), gas exchange parameters were measured using a portable IRGA (Infra-Red Gas Analyzer, Hoddesdon, UK) before harvesting the plants. For different gas exchange parameters such as transpiration rate (T*r*), stomatal conductance (G*s*), net photosynthesis (P*n*), and water use efficiency (W*i*), 3 leaves were selected randomly from each treatment and from 3 different plants of a single treatment. Moreover, we selected 3 different leaves from each treatment, which were alike (means no difference in age were observed).

The content of malondialdehyde (MDA) and hydrogen peroxide (H_2_O_2_) was measured by following the method of Heath and Packer [24] and Jana and Choudhuri [25], respectively. The stress-induced electrolyte leakage (EL) was measured by the method presented by Dionisio-Sese and Tobita’s [26] by using the following formula:

EL = (EC_1_/EC_2_) × 100

The activities of Antioxidants such SOD, POD, APX and CAT measured by following the method of Chen and Pan [27], Sakharov and Ardila [28], Aebi [29] and Nakano and Asada [30] respectively.

Plant samples were vigilantly digested via the di-acid (HNO_3_-HClO_4_) technique. 0.5 g dry sample (3 replications for each treatment) of roots and shoots of the plants were taken into the flask, having 10 mL of HNO_3_-HClO_4_ (3:1, *v*:*v*), this collection was then retained overnight. The final digestion of these plants’ samples was completed after the addition of HNO_3_ (5 mL) and then placed on the hot plate for complete digestion, as described by Rehman et al. [31]. Atomic absorption spectrophotometer (AAS) was used to investigate the correct amount of Cr and Zn in shoots and roots of the plant.

### 2.5. Statistical Analysis

All the data were assayed with the help of SPSS software through a multivariate post hoc test, in order to determine the interaction among significant values. One-way analysis of variance (ANOVA) was used to assess the significance of the variations of Cr among the different plant parts, followed by HSD tests. Considerable variation among different observed values was calculated at *p* < 0.05 and displayed with various letters. The graphical presentation was carried out using Origin Pro (2017) (Systat Software Inc., San Jose, CA, USA). The Pearson correlation coefficients between the measured variables of *B. napus* were also calculated. The plots of principal component analysis on rapeseed parameters were carried out by using the Rstudio software. Moreover, heatmap was also presented for the different variables of *B. napus* by using the Rstudio software.

## 3. Results

### 3.1. Effect of Foliar Application of Zn-lys on Plant Growth and Biomass under Different Levels of Tannery Wastewater

Data regarding different morphological parameters of *B. napus* are presented in Table 3. According to the results, the toxic content of Cr in the wastewater caused a significant decrease in all morphological traits of *B. napus* studied in this study (Table 3). Compared to the control, maximum decreased in plant height, the number of leaves, leaf area, root length, fresh root weight, root dry weight, fresh leaf weight, dry leaf weight by 68.6%, 54.5%, 84.5%, 38.4%, 89.7%, 89.5%, 78.6%, and 82.3%, respectively, in T6 plants (irrigated 100% tannery wastewater without the application of Zn-lys). However, the application of Zn-lys increased plant height, the number of leaves, leaf area, root length, fresh root weight, root dry weight, fresh leaf weight, dry leaf weight at all levels of tannery wastewater (33%, 66%, and 100%) in the soil and increased by 10%, 20%, 11.8%, 25%, 258.2%, 17.1%, 16.7%, and 17.1% at 100% addition of tannery wastewater treatment in the soil with the application of Zn-lys compared to 100% addition of tannery wastewater treatment in the soil without the application of Zn-lys. Although the data regarding (T7) were statistically non-significant with (T6), which was showing that the application of Zn-lys at 100% wastewater level did not significantly affect the plant growth and biomass in *B. napus*.

### 3.2. Effect of Foliar Application of Zn-lys on Photosynthetic Pigments and Gas Exchange Attributes under Different Levels of Tannery Wastewater

**Figure 1:**
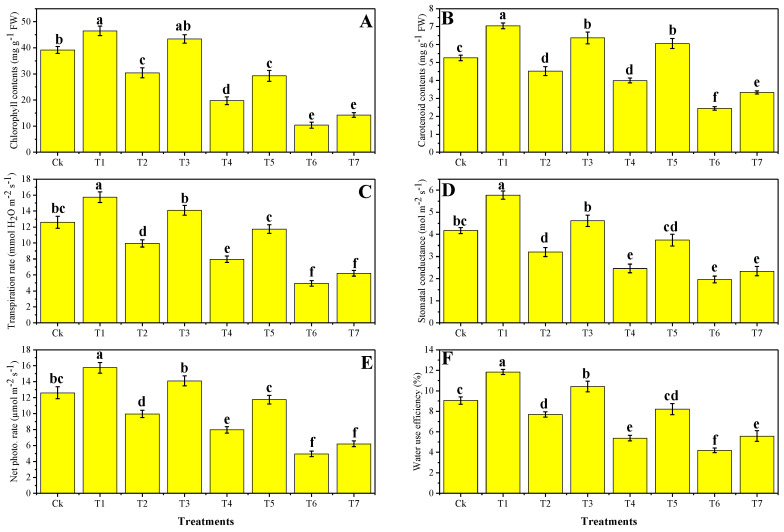
The effect of different levels of tannery wastewater on total chlorophyll contents (**A**), carotenoid contents (**B**), transpiration rate (**C**), stomatal conductance (**D**), net photosynthesis (**E**) and water use efficiency (**F**) under the same concentration of Zinc-lysine application in *B. napus*. Values are demonstrated as means of three replicates along with the standard deviation (SD; *n* = 3). One-way ANOVA was performed, and the mean differences were tested by HSD (*p* < 0.05). Different lowercase letters on the error bars indicate a significant difference between the treatments. Different treatments used in the figures are as follows: Ck (without irrigation with wastewater + 0 mg/L Zn-lysine), T1 (without irrigation with wastewater + 10 mg/L Zn-lysine), T2 (33% irrigation with wastewater + 0 mg/L Zn-lysine), T3 (33% irrigation with wastewater + 10 mg/L Zn-lysine), T4 (66% irrigation with wastewater + 0 mg/L Zn-lysine), T5 (66% irrigation with wastewater + 10 mg/L Zn-lysine), T6 (100% irrigation with wastewater + 0 mg/L Zn-lysine), and T7 (100% irrigation with wastewater + 10 mg/L Zn-lysine).

In the current experiment, we have noticed that total chlorophyll content (chl *a* + chl *b*), carotenoid content, and various gas exchange parameters were decreased significantly (*p* < 0.05) in *B. napus*, when irrigated with different levels of tannery wastewater with the application of Zn-lys. The increasing level of tannery wastewater (33%, 66%, and 100%) in the soil decreased photosynthetic pigments and gas exchange parameters of *B. napus* when compared to the control treatment. The data regarding these parameters are presented in Figure 1. According to the given data, we have noticed that the maximum content of the total chlorophyll (chl *a* + chl *b*), carotenoid, transpiration rate (T*r*), stomatal conductance (G*s*), net photosynthesis (P*n*), and water use efficiency (W*i*) were decreased in T6 treated plants by 74.4%, 53.8%, 32%, 52.9%, 61.1%, and 53.4%, respectively, compared to the plants grown in the control treatment (without the addition of tannery wastewater and without the application of Zn-lys). Although, the exogenous supplementation of Zn-lys increased chl, carot, T*r*, G*s*, P*n*, and W*i* by 37.5%, 36.4%, 6.5%, 40%, 5.7%, and 126.3%, respectively, in T7 plants when compared to the plants grown in T6 (100% wastewater without application of Zn-lys). Although the data regarding (T7) were statistically non-significant with (T6), which was showing that the application of Zn-lys at 100% wastewater level did not significantly affect photosynthetic pigments and gas exchange attributes in *B. napus*.

### 3.3. Effect of Foliar Application of Zn-lys on Oxidative Stress and Antioxidant Response under Different Levels of Tannery Wastewater

**Figure 2:**
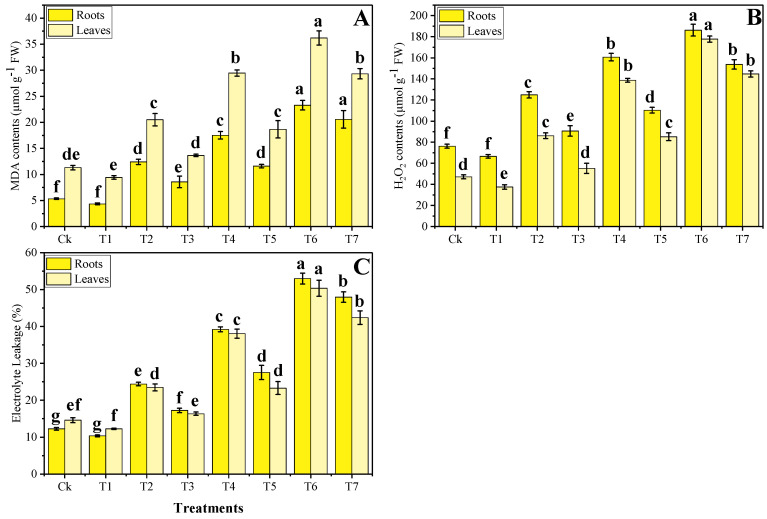
The effect of different levels of tannery wastewater on MDA contents (**A**) H_2_O_2_ contents (**B**) and EL leakage (**C**) in the roots and leaves of *B. napus* under the same concentrations of Zinc-lysine application. Values are demonstrated as means of the three replicates along with the standard deviation (SD; *n* = 3). One-way ANOVA was performed, and mean differences were tested by HSD (*p* < 0.05). Different lowercase letters on the error bars indicate a significant difference between the treatments. Different treatments used in the figures are as follows: Ck (without irrigation with wastewater + 0 mg/L Zn-lysine), T1 (without irrigation with wastewater + 10 mg/L Zn-lysine), T2 (33% irrigation with wastewater + 0 mg/L Zn-lysine), T3 (33% irrigation with wastewater + 10 mg/L Zn-lysine), T4 (66% irrigation with wastewater + 0 mg/L Zn-lysine), T5 (66% irrigation with wastewater + 10 mg/L Zn-lysine), T6 (100% irrigation with wastewater + 0 mg/L Zn-lysine), and T7 (100% irrigation with wastewater + 10 mg/L Zn-lysine).

**Figure 3:**
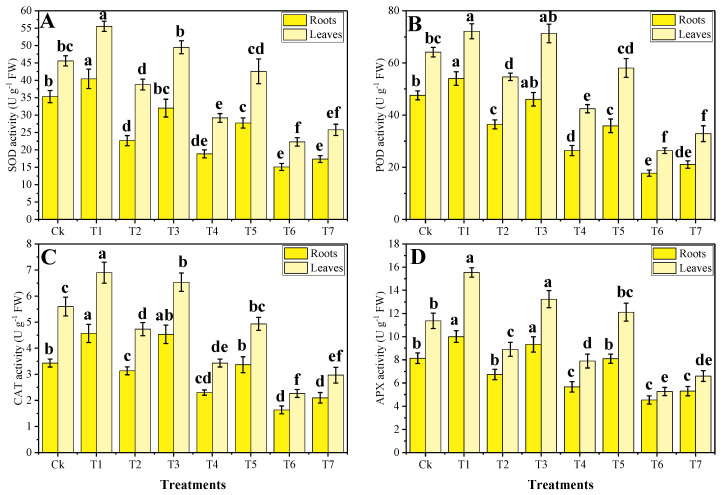
The effect of different levels of tannery wastewater on the activities of SOD (**A**), POD (**B**), CAT (**C**) and APX (**D**) in the roots and leaves under the same concentrations of Zinc-lysine application in *B. napus*. Values are demonstrated as the means of three replicates along with the standard deviation (SD; *n* = 3). One-way ANOVA was performed, and the mean differences were tested by HSD (*p* < 0.05). Different lowercase letters on the error bars indicate a significant difference between the treatments. Different treatments used in the figures are as follow: Ck (without irrigation with wastewater + 0 mg/L Zn-lysine), T1 (without irrigation with wastewater + 10 mg/L Zn-lysine), T2 (33% irrigation with wastewater + 0 mg/L Zn-lysine), T3 (33% irrigation with wastewater + 10 mg/L Zn-lysine), T4 (66% irrigation with wastewater + 0 mg/L Zn-lysine), T5 (66% irrigation with wastewater + 10 mg/L Zn-lysine), T6 (100% irrigation with wastewater + 0 mg/L Zn-lysine) and T7 (100% irrigation with wastewater + 10 mg/L Zn-lysine).

Increased in the content of MDA, H_2_O_2_ initiation, and EL (%) indicating that a high content of metal in the soil, used for the pot experiment, caused oxidative damage in *B. napus*. Although, activities of various antioxidant enzymes were initially increased due to Cr toxicity and then decreased at extreme concentration of Cr concentration in the soil. The data regarding oxidative stress and antioxidant response are presented in Figure 2 and Figure 3, respectively. In our results, we analyzed roots and shoots differently for their statistics. Compared to the control treatment, the maximum content of MDA, H_2_O_2_ initiation, and EL (%) were showed in T6 plants, which were increased by 4.3, 2.5, and 4 times, respectively, in the roots and 3.1, 3.7, and 3.4 times, respectively. While the exogenous application of Zn-lys decreased the content of MDA, H_2_O_2_ initiation, and EL (%) in the roots as well as in the leaves of *B. napus*. Similarly, activities of antioxidants initially increased (33% of wastewater in the soil) compared to the control treatment, but further addition of wastewater in the soil caused a significant decrease in antioxidant activities. Compared to the plants grown in the control treatment, the maximum activities of SOD, POD, CAT, and APX were increased by 14.2%, 12.5%, 23%, and 30.3%, respectively, in the roots and 22.2%, 19.3%, 38.8%, and 23.2%, respectively, in the leaves in T1 plants. Our results also showed that the foliar application of Zn-lys increased the activity of antioxidant enzymes when compared with those plants, which were grown without the foliar application of Zn-lys in all treatments of wastewater, including the control treatment (Figure 3). Moreover, the plants grown in the T7 showed a non-significant decrease in the activities of antioxidants (in both roots and shoots of the plants), compared with the plants grown in T6, suggesting that application of Zn-lys showed a non-significant decrease in antioxidants when grown in 100% addition of tannery wastewater in the soil.

### 3.4. Effect of Foliar Application of Zn-lys on Uptake and Accumulation of Cr and Zn under Different Levels of Tannery Wastewater

**Figure 4:**
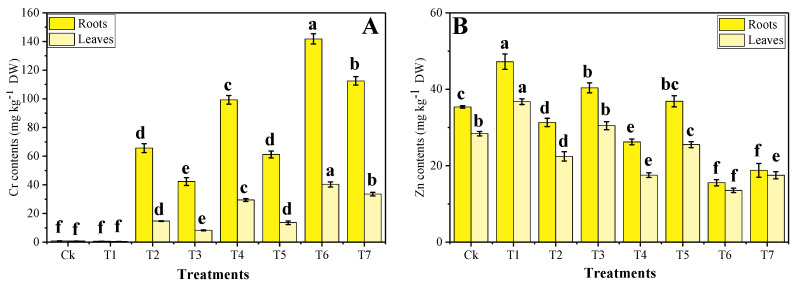
The effect of different levels of tannery wastewater on the uptake of Cr (**A**) and Zn (**B**) content in the roots and leaves under the same concentrations of Zinc-lysine application in *B. napus*. Values are demonstrated as the means of three replicates along with standard deviation (SD; *n* = 3). One-way ANOVA was performed, and the mean differences were tested by HSD (*p* < 0.05). Different lowercase letters on the error bars indicate a significant difference between the treatments. Different treatments used in the figures are as follow: Ck (without irrigation with wastewater + 0 mg/L Zn-lysine), T1 (without irrigation with wastewater + 10 mg/L Zn-lysine), T2 (33% irrigation with wastewater + 0 mg/L Zn-lysine), T3 (33% irrigation with wastewater + 10 mg/L Zn-lysine), T4 (66% irrigation with wastewater + 0 mg/L Zn-lysine), T5 (66% irrigation with wastewater + 10 mg/L Zn-lysine), T6 (100% irrigation with wastewater + 0 mg/L Zn-lysine) and T7 (100% irrigation with wastewater + 10 mg/L Zn-lysine).

The results regarding Cr and Zn uptake in different organs (roots and shoots) of *B. napus* were also determined in this study. In our results, we analyzed roots and shoots differently for their statistics with one-way ANOVA. The data regarding Cr and Zn uptake are presented in Figure 4 and suggested that increasing levels of wastewater in the soil caused a significant increase in Cr uptake in all organs of the plants and a decrease in the content of Zn in all organs of the plant. However, Zn was accumulated at a higher concentration up to a level (33%). At the same time, further addition of wastewater caused a significant decrease in Zn content in all organs of the *B. napus* (Figure 4). According to the results, the maximum content of Cr was observed in T6 plants, which were 141 mg kg^−^^1^ in the roots and 40 mg kg^−^^1^ in the shoots of *B. napus*. In contrast, Zn content was found to be maximum in T1 plants 47 mg kg^−^^1^ in the roots and 37 mg kg^−^^1^ in the shoots compared with the plants grown without the application of Zn-lys without the addition of tannery wastewater in the soil. However, the addition of tannery wastewater (33%, 66%, and 100%) in the soil, significantly decreased the Zn content in the roots and shoots of the plants, when compared with the plants grown without the application of Zn-lys without the addition of tannery wastewater in the soil. In contrast, foliar application of Zn-lys significantly decreased Cr content while increasing Zn content in the roots and shoots of the plants compared with those treatments in which plants were grown without the application of Zn-lys. However, non-significant results were also obtained in the Zn content of roots in T6 and T7 plants.

### 3.5. Correlation between Different Parameters Studied in This Experiment

**Figure 5:**
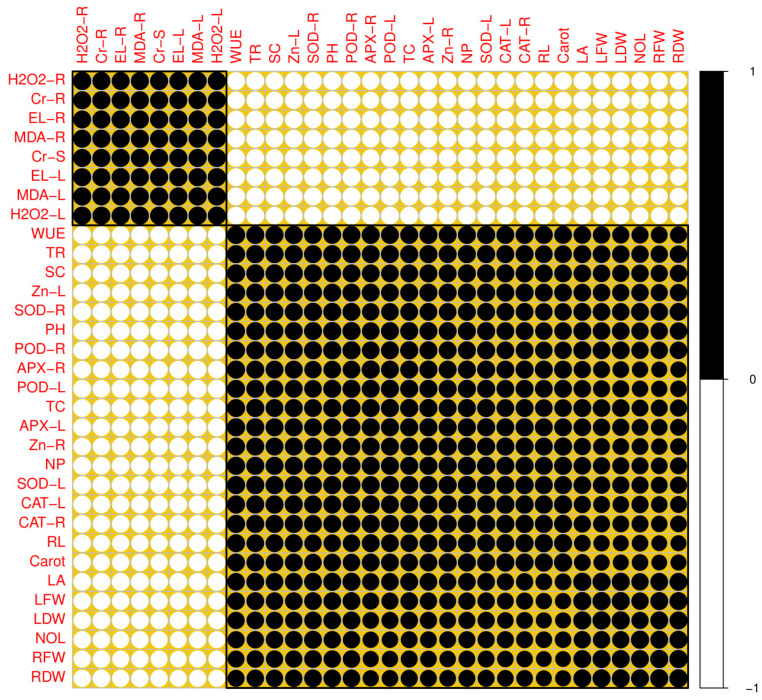
Correlation between different attributes studied in this study. H2O2-R (H_2_O_2_ initiation in roots), CR-R (Cr content in roots), EL-R (electrolyte leakage in roots), MDA-R (MDA content in roots), CR-S (Cr content in shoots), EL-L (electrolyte leakage in leaves), MDA-L (MDA content in leaves), H2O2-L (H_2_O_2_ initiation in leaves), Zn-R (zinc content in roots), NP (net photosynthesis), SOD-L (SOD activity in leaves), WUE (water use efficiency), TR (transpiration rate), SC (stomatal conductance), Zn-L (zinc content in leaves), SOD-R (SOD activity in roots), PH (plant height), POD-R (POD activity in roots), CAT-L (CAT activity in leaves), CAT-R (CAT activity in roots), RL (root length), Carot (carotenoid content), APX-R (APX activity in roots), POD-L (POD activity in leaves), TC (total chlorophyll content), APX-L (APX activity in leaves), LA (leaf area), LFW (leaves fresh weight), LDW (leaves dry weight), NOL (number of leaves), RFW (root fresh weight), RDW (root dry weight).

A correlogram was analyzed to quantify the relationship between different morpho-physiological attributes with Cr and Zn uptake by the plant organs (Figure 5). According to the results, it was suggested that Cr content in the roots was positively correlated with Cr concentration in the shoots and oxidative stress indicators while negatively correlated with plant growth and biomass, physiological traits and antioxidative enzymes, and Zn content in all organs of the plants. Similarly, Cr concentration in the shoots was positively correlated with Cr concentration in the roots, oxidative stress indicators while negatively correlated with all other morpho-physiological traits and Zn uptake. Moreover, the correlation graph also showed that Cr content in the roots was significantly increased with the Cr content in the shoots and also oxidative stress indicators and vice versa. However, Cr content in the roots was significantly decreased plant morphological and physiological attributes studied in this experiment.

**Figure 6:**
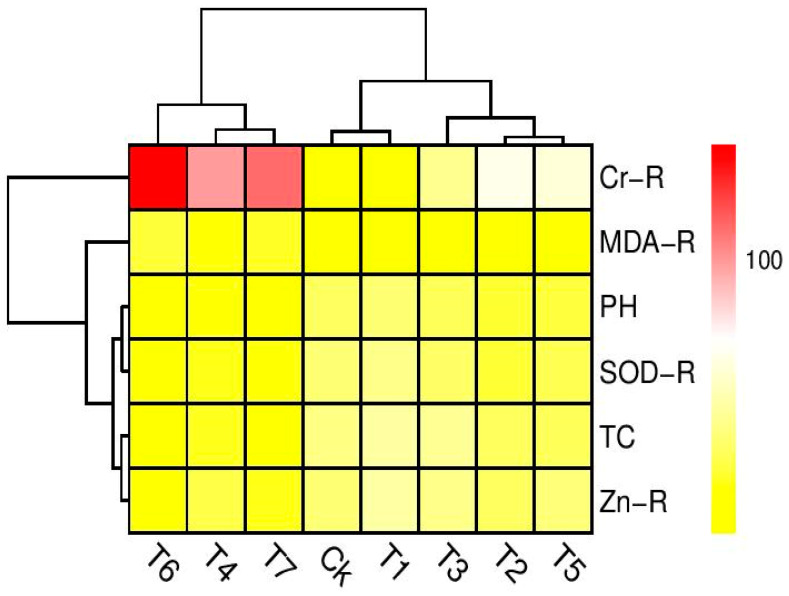
Heatmap histogram correlation between different parameters studied in this experiment. (Ck) Cr 0%, Zn-lys 0 mg L^−1^ (T1) Cr 0%, Zn-lys 10 mg L^−1^ (T2) Cr 33%, Zn-lys 0 mg L^−1^ (T3) Cr 33%, Zn-lys 10 mg L^−1^ (T4) Cr 66%, Zn-lys 0 mg L^−1^ (T5) Cr 66%, Zn-lys 10 mg L^−1^ (T6) Cr 100%, Zn-lys 0 mg L^−1^ (T7) Cr 100%, Zn-lys 10 mg L^−1^. The abbreviations are as follow: CR-R (Cr content in roots), MDA-R (MDA content in roots), PH (plant height), SOD-R (SOD activity in roots), TC (total chlorophyll content), and Zn-R (zinc content in roots).

A heatmap-histogram analysis was also constructed to explore the relationship between the different growth and Cr uptake attributes (Figure 6). Only significant differences were observed in the Cr uptake in T4, T6, and T7, while all other selected traits were showing non-significant differences with all treatments that were used in this study. The color yellow in Figure 6 indicates the non-significant results of our study, and the red color showed significant results in this figure. This histogram depicted a clear difference between the Cr uptake abilities and growth attributes of *B. napus*.

### 3.6. Principal Component Analysis

**Figure 7:**
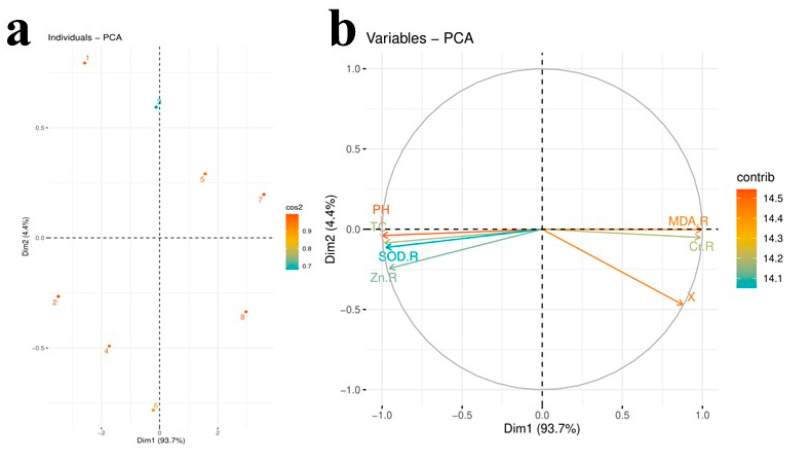
Score (**a**) and loading plots (**b**) of principal component analysis (PCA) on different studied attributes of *B. napus* grown in tannery wastewater soil. Score plot represents separation of treatments (1) Cr 0%, Zn-lys 0 mg L^−1^ (2) Cr 0%, Zn-lys 10 mg L^−1^ (3) Cr 33%, Zn-lys 0 mg L^−1^ (4) Cr 33%, Zn-lys 10 mg L^−1^ (5) Cr 66%, Zn-lys 0 mg L^−1^ (6) Cr 66%, Zn-lys 10 mg L^−1^ (7) Cr 100%, Zn-lys 0 mg L^−1^ (8) Cr 100%, Zn-lys 10 mg L^−1^. The abbreviations are as follows: Zn-R (zinc content in roots), TC (total chlorophyll content), PH (plant height), SOD-R (SOD activity in roots), CR-R (Cr content in roots), and MDA-R (MDA content in roots).

The scores and loading plots of PCA to check the effect of different levels of tannery wastewater with the exogenous application of Zn-lys on some selected attributes of *B. napus* plants are presented in Figure 7. Of all the main components, the first two components-Dim1 and Dim2-comprised more than 98% of the whole database and make up the largest portion of all components (Figure 7a). Among this, Dim1 contributed 93.7%, and Dim2 contributed 4.4% of the whole dataset. According to the results, all the respected treatments were dispersed successfully in the whole dataset (Figure 7a). The distribution of all the components in the dataset gave a clear indication that Cr toxicity in the soil significantly affected all the treatments used in this study with the exogenous application of Zn-lys. The control (1) treatment was most displaced from all other treatments of tannery wastewater in the soil, indicating that Cr toxicity in the soil significantly affected morpho-physiological traits of *B. napus* plants with the exogenous application of Zn-lys. However, Figure 7b shows that MDA and Cr content in the roots were positively correlated in the dataset from all the variables. In contrast, SOD activity in the roots, Zn content in the roots, plant height, and total chlorophyll content in the leaves were negatively correlated in PCA with all other variables (Figure 7b).

## 4. Discussion

The harmful effects of heavy metals on food safety and marketability, plant growth because of plant-toxicity, and the ecosystem health of soil organisms cause serious metal accumulation in soils in agricultural production [32,33]. Like all living organisms, plants are very often both susceptible to the shortage and abundance of the available heavy metal ions as the key micronutrient, although they are also very toxic to normal plant growth and development at higher concentrations and even more ions such as Cr [7,10]. In our previous study, we also observed that the toxic concentration of Cu in the nutrient solution changed growth and other morphological attributes in the 40-day-old *B. napus* seedlings [18]. Although, in the current study, increasing levels of wastewater (33%, 66%, and 100%) in the soil displayed inhibitory effects on all morphological traits of the *B. napus* by declining different parameters of plant growth and biomass when compared to the control treatment (Table 1). Under the toxic level of Cr in the soil, the similar findings were showed by Li et al. [16] and Zaheer et al. [14], when they studied *B. napus* under different levels of Cr in the soil. This is because Cr can accumulate quickly in different organs of the plants, as suggested by Hussain et al. [9], which induced toxicity in the plants. The results from the rapid uptake of Cr content by the plant organs caused stunned growth and declined biomass in all parts of the plants [34]. Furthermore, it was also noticed that a very high concentration of Cr in the soil decreased some essential nutrients in the soil, which are important for normal growth and development of a plant body [10,34].

Photosynthetic pigments and gas exchange attributes were also affected with the addition of wastewater (33%, 66%, and 100%) in the soil (Figure 1), and it was observed that Cr toxicity in the soil significantly decreased these attributes in *B. napus*. Reduction in chlorophyll concentrations may lead to the removal of Mg needed for chlorophyll biosynthesis and/or ultra-structural modification to metal toxicity of chloroplast [18,35]. In addition to that, Cr toxicity influences and decreases the composition of photosynthetic pigments, which has already been shown by Yu et al. [36], PSII photochemical activity. In the leaves of *B. napus*, a remarkable decrease in photosynthetic pigments and gas exchange parameters were also noticed by Hasanuzzaman et al. [37] under Cd stress and Zhang et al. [17] under Cu stress environment. Our results coincide with the findings of Yasmeen et al. [38], which also noticed that the excess Cr concentration in the soil could cause a significant decrease in photosynthesis of the plants, including *B. napus*. Moreover, the disturbance in the transport of the water from the roots to the aboveground parts of the plants resulted in a decline in stomatal conductance and transpiration rate, which ultimately destroy the gas exchange parameters of the plants [39].

The high concentration of pollutants (heavy metals) in the soil induce oxidative stress [2], however, plants under Cr stress face severe oxidative damage owing to the overproduction of ROS and electrolyte leakage [9,16]. Phytotoxicity of Cr damaged many membrane-bounded organelles of the plant’s results in oxidative stress in the plants and also a factor in lowering the biomass of the plants [34]. The extra production of ROS is extremely toxic for the plants and can cause the death of the plant, however, plants have an antioxidative defense system to scavenge these ROS [33,40]. *B. napus* is resistant to heavy metal stress but showed much higher production of O^−2^, MDA, and H_2_O_2_ when grown in metal-polluted soil [16,18]. However, several studies also suggested that *B. napus* has a very strong defense system (antioxidants), which ultimately increased their activities to protect the plant against the metal stress environment [2,13,14]. In the current study, Cr toxicity caused oxidative stress in the *B. napus* by increasing malondialdehyde (MDA) content in the roots and leaves of the plants (Figure 2). This is because Cr toxicity disrupts the electron transport chain, producing much higher levels of H_2_O_2_ and O^−2^ radicals and increasing electrolyte leakage afterwards [10]. Extremely high contents of Cr in the plants can decrease the activities of antioxidant enzymes, which was also noticed in the current experiment with the addition of 66% and 100% tannery wastewater in the soil, which decreased the activities of various antioxidant enzymes in B. napus significantly (Figure 3). This alteration in the activities of different antioxidants might be due to the alterations in different genetic expressions and functions of some important proteins, which take part in this mechanism [8]. The decrease in the activities of antioxidants under elevating the level of Pb (300 mg kg^−1^ and 600 mg kg^−1^) in the soil was also observed in the castor beans [41].

The absorption of heavy metals from the soil and transportation to various plant parts depends primarily on metal supplies and growth conditions. Heavy metals from the soil are absorbed by the root cell membrane and transferred and detoxified throughout the plant [42,43]. Plants with a higher Cr accumulation are not able to absorb the required range of essential nutrients, which will eventually lead to normal growth and development differences due to distorting photosynthesis and structural distortions [36]. In the current experiment, our objective is to reduce Cr content in different organs of the *B. napus* using a micronutrient-amino chelator. However, a very large amount of wastewater in the soil increased the Cr content in the various parts of the plant (Figure 4). There are several studies that suggest that increased soil Cr levels have caused a significant increase in Cr uptake and accumulation in different parts of the plants [34,44]. However, a reduction in the Zn uptake (which is an essential micronutrient) might be linked with the inability of the roots to absorb it from the soil under the high content of Cr in the soil [9]. It was also noticed that plants, when cultivated with tannery wastewater (which has a high content of toxic pollutants such as Cr), are unable to absorb/uptake a vital range of essential nutrients, which not only reduced micronutrients in the plant organs but also reduced different morpho-physiological attributes in the plants [6].

Various plant growth hormones have been widely used recently to mitigate oxidative stress and prompt plant growth and composition in different plant species when grown in metal-polluted soil [1,18,40]. Although, the usage of micronutrients with the amino acid complex is an incredibly new idea and showed beneficial results such as improved plant growth and composition and restrict the plant for uptake/accumulate toxic content in their body parts. There is also some literature on various plant species, which, when grown on metal contaminated soil, alleviates metal toxicity and improves plant growth and biomass [12,14,45]. In our previous study, we also noticed that irrigation with wastewater caused metal toxicity due to a large amount of Cr content in the soil, while an application with Zn-lys alleviates metal toxicity by increasing plant growth and biomass in spinach plants [3]. The reduced Cr concentration in different parts of *B. napus* could be due to the higher Zn concentration in plants, as Zaheer et al. [3] previously reported antagonistic effects of Cr and Zn. This is because the application of Zn-lys gives plants space to increase/absorb essential nutrients from the soil and a more secure option for plants in the stressful environment. In addition, a large amount of amino acid in a plant’s various organs plays a crucial role in reducing the movement of metal pollutants in plants by forming different complexes [45]. Amino acids also take part in the ultra-structure constitution, which is also another factor in improving plant growth and photosynthesis in the plants [46]. Exogenous supplementation of Zn-lys has shown the ability to boost up the W*i* of the plant by enhancing the nutrient uptake, which as a result increases plant growth by governing the processes of transpiration and photosynthesis [9,47]. Our results also showed that the application of Zn-lys reduced oxidative stress in the plants by enhancing the activities of different enzymatic antioxidants (Figure 2 and Figure 3). The decrease in oxidative stress in *B. napus* might be due to the accumulation of a large amount of amino acids, which have the ability to scavenge increased production of ROS, as suggested by Teixeira et al. [48]. In the current experiment, exogenous supplementation of Zn-lys caused a significant increase in Zn content while a noticeable decrease in the Cr content in all organs (roots and shoots) of the plants was observed (Figure 4). This is because of external application of Zn-lys, which not only increased Zn content in roots and shoots of the plants and also decreased Cr content because amino acids have the ability to form complexes with extra heavy metals and restrict their mobility in the plant organs [12,45].

## 5. Conclusions

Irrigation of *B. napus* with tannery wastewater is dangerous because it may contain toxic pollutants (such as Cr), which significantly affect its growth and yield. Although, phytotoxicity of Cr also decreased their photosynthetic pigments and gas exchange parameters and induced oxidative damage due to the overproduction of ROS in the cell/tissues of the plants. Moreover, Cr toxicity also decreased essential nutrients (such as Zn) in the plant organs, which is essential for normal growth and development of the plant body. The exogenous application of Zn-lys can reduce the phytotoxicity of Cr by increasing morphological characteristics and activity of antioxidant enzymes, which helps to mitigate oxidative stress in plants. Taken together, foliar supplementation of Zn-lys amoriates Cr stress in *B. napus* by improving plant growth and yield. Therefore, Zn-lys chelate can be efficiently used for improving *B. napus* growth and Zn contents while reducing Cr concentration in plants in a Zn deficient and Cr-contaminated soil.

## Figures and Tables

**Table 1:** Physicochemical properties of loam sand used in the pot experiment.

Texture	Clay Loam
Silt	12.9
Sand	63.4
Clay	22.3
pH (H_2_O mixture)	7.1
Electrical conductivity (dS m^−1^)	3.83
Cation exchange capacity (cmol kg^−1^) 4.9	4.89
Soluble CO_3_^−2^ (mmol L^−1^)	0.87
Soluble HCO_3_ (mmol L^−1^)	3.78
Soluble Cl^−^ (mmol L^−1^)	6.31
Soluble Ca^2+^ + Mg^2+^ (mmol L^−1^)	15.89
Organic matter (%)	0.49
Ni (mg kg^−1^)	0.21
Cu (mg kg^−1^)	0.35
Zn (mg kg^−1^)	0.84
Cr (mg kg^−1^)	0.24

**Table 2:** Characteristics of tannery wastewater used for irrigation of the soil used in the pot experiment.

Parameters	Values	Permissible Limits *
EC (dS m^−1^)	1.41	<1.5
Sodium absorption rate (mmol L^−1^)^1/2^	4.02	<7.5
Residual sodium carbonate (mmol c L^−1^)	2.24	<2.0
Ni (mg L^−1^)	0.09	0.20
Cd (mg L^−1^)	0.04	0.01
Pb (mg L^−1^)	1.24	5.0
Co (mg L^−1^)	0.02	0.05
Cr (mg L^−1^)	4.03	0.10
Zn (mg L^−1^)	1.95	2.00

* Ayers and Westcot [22].

**Table 3:** Effect of different levels of tannery wastewater on plant growth and biomass under the same concentrations of Zinc-lysine application in *B. napus.*

Treatments	Plant Height (cm)	Number of Leaves	Leaf Area (cm^2^)	Root Length (cm)	Root Fresh Weight (mg)	Root Dry Weight (mg)	Leaf Fresh Weight (mg)	Leaf Dry Weight (mg)
Ck	31 ± 0.6ab	11 ± 1ab	110 ± 7ab	13 ± 0.4cd	4025 ± 55b	837 ± 36b	19,336 ± 740b	2457 ± 50b
T1	34 ± 1.0a	13 ± 1a	126 ± 5a	17 ± 1a	5038 ± 70a	999 ± 46a	23,675 ± 1200a	2658 ± 64a
T2	22 ± 1.6de	6 ± 0.6bc	72 ± 5d	12 ± 0.6de	1661 ± 57d	334 ± 13d	11,991 ± 467d	1573 ± 56d
T3	29 ± 1.2bc	8 ± 0.6b	91 ± 4c	15 ± 0.7ab	2191 ± 64c	470 ± 18c	15,599 ± 561c	1804 ± 50c
T4	17 ± 0.6ef	6 ± 1c	38 ± 3f	10 ± 0.3e	798 ± 37f	198 ± 12e	5005 ± 100f	729 ± 30f
T5	24 ± 1.7cd	8 ± 0.6b	53 ± 3e	14 ± 0.8bc	1179 ± 53e	292 ± 19d	6872 ± 170e	909 ± 30e
T6	10 ± 0.6g	5 ± 0.6c	17 ± 1g	8 ± 0.5f	414 ± 13g	88 ± 6f	3142 ± 70g	433 ± 24g
T7	11 ± 2.8fg	6 ± 0.6bc	19 ± 3g	10 ± 0.6e	505 ± 19g	103 ± 10f	3665 ± 75fg	507 ± 28g

Values are demonstrated as means of three replicates along with standard deviation (SD; *n* = 3). One-way ANOVA was performed and mean differences were tested by highest significant deviation (HSD) at *p* < 0.05. Different lowercase letters indicate a significant difference between the treatments. Different abbreviations used in the table are as follows: Ck (without irrigation with wastewater + 0 mg/L Zn-lysine), T1 (without irrigation with wastewater + 10 mg/L Zn-lysine), T2 (33% irrigation with wastewater + 0 mg/L Zn-lysine), T3 (33% irrigation with wastewater + 10 mg/L Zn-lysine), T4 (66% irrigation with wastewater + 0 mg/L Zn-lysine), T5 (66% irrigation with wastewater + 10 mg/L Zn-lysine), T6 (100% irrigation with wastewater + 0 mg/L Zn-lysine) and T7 (100% irrigation with wastewater + 10 mg/L Zn-lysine).
